# Sleep Duration Modifies the Association of Overtime Work With Risk of Developing Type 2 Diabetes: Japan Epidemiology Collaboration on Occupational Health Study

**DOI:** 10.2188/jea.JE20170024

**Published:** 2018-07-05

**Authors:** Keisuke Kuwahara, Teppei Imai, Toshiaki Miyamoto, Takeshi Kochi, Masafumi Eguchi, Akiko Nishihara, Tohru Nakagawa, Shuichiro Yamamoto, Toru Honda, Isamu Kabe, Tetsuya Mizoue, Seitaro Dohi

**Affiliations:** 1 Department of Epidemiology and Prevention, Bureau of International Health Cooperation, National Center for Global Health and Medicine, Tokyo, Japan; 2 Teikyo University Graduate School of Public Health, Tokyo, Japan; 3 Azbil Corporation, Tokyo, Japan; 4 Nippon Steel & Sumitomo Metal Corporation Kimitsu Works, Chiba, Japan; 5 Furukawa Electric Co., Ltd., Tokyo, Japan; 6 Hitachi, Ltd., Ibaraki, Japan; 7 Mitsui Chemicals, Inc., Tokyo, Japan

**Keywords:** long working hours, sleep habits, Asians, cohort study, prevention

## Abstract

**Background:**

Evidence linking working hours and the risk of type 2 diabetes mellitus (T2DM) is limited and inconsistent in Asian populations. No study has addressed the combined association of long working hours and sleep deprivation on T2DM risk. We investigated the association of baseline overtime work with T2DM risk and assessed whether sleep duration modified the effect among Japanese.

**Methods:**

Participants were Japanese employees (28,489 men and 4,561 women) aged 30–64 years who reported overtime hours and had no history of diabetes at baseline (mostly in 2008). They were followed up until March 2014. New-onset T2DM was identified using subsequent checkup data, including measurement of fasting/random plasma glucose, glycated hemoglobin, and self-report of medical treatment. Hazard ratios (HRs) of T2DM were estimated using Cox regression analysis. The combined association of sleep duration and working hours was examined in a subgroup of workers (*n* = 27,590).

**Results:**

During a mean follow-up period of 4.5 years, 1,975 adults developed T2DM. Overtime work was not materially associated with T2DM risk. In subgroup analysis, however, long working hours combined with insufficient sleep were associated with a significantly higher risk of T2DM (HR 1.42; 95% CI, 1.11–1.83), whereas long working hours with sufficient sleep were not (HR 0.99; 95% CI, 0.88–1.11) compared with the reference (<45 hours of overtime with sufficient sleep).

**Conclusions:**

Sleep duration modified the association of overtime work with the risk of developing T2DM. Further investigations to elucidate the long-term effect of long working hours on glucose metabolism are warranted.

## INTRODUCTION

Worldwide, people work for long hours (eg, ≥48 hours per week).^1^ The effect of this on health, especially cardiovascular disease, has been investigated.^2^ Recently, the effect of working long hours on glucose metabolism has gained much attention, although findings are inconsistent. A meta-analysis of cohort studies, mainly from Europe and the United States,^3^ showed no association of working hours with the risk of type 2 diabetes mellitus (T2DM). In Asia, epidemiological evidence is scarce and conflicting.^4^^–^^7^

Given that the prevalence of working long hours in Asian countries is higher (20–50%) than in European countries (10–20%),^1^ the effect of working long hours on the development of T2DM should be clarified in Asia. Previous Asian studies, however, are limited by relatively small sample sizes (1,000–3,000 participants)^4^^,^^5^^,^^7^ and the use of simple categorization of working hours into two or three categories.^4^^–^^6^ A Japanese study reported a higher risk of myocardial infarction among adults with long working hours and short sleep duration, suggesting that sleep may act as an effect modifier.^8^ However, this has not been investigated in terms of the risk of developing T2DM.

We recently reported a cross-sectional association of working hours with having diabetes in a large cohort of Japanese workers.^9^ In the present study, we investigated the prospective association between overtime work, including a category of extremely long hours, and risk of T2DM in the same cohort. We also examined the overtime work-T2DM risk association stratified by hours of sleep in a sub-cohort for which the data on sleep were available.

## METHODS

### Study procedure

This cohort study was performed using data on annual health checkups in the sub-cohort of Japan Epidemiology Collaboration on Occupational Health (J-ECOH) Study,^10^^,^^11^ an ongoing, multi-center epidemiologic study among workers from more than 10 companies in Japan. Workers in Japan are obliged to have a health checkup at least once per year under the Industrial Safety and Health Act. Before data collection, the conduct of the J-ECOH Study was announced in each of the participating companies using posters to explain the purpose and procedure of the study. The need for participants to provide informed consent for this study was waived. This procedure conforms to the Japanese Ethical Guidelines for Epidemiological Research. The study protocol was approved by the Ethics Committee of the National Center for Global Health and Medicine, Japan.

We extracted data of 52,504 workers (including 8,229 women) at ages 30–64 years who underwent health checkups mainly in 2008 at four companies, and in 2010 at one company, where data on overtime work were available. We followed participants until March 2014.

### Participants

Of the initial 52,504 workers whose data were extracted, we excluded 16,147 at baseline as follows: 7,316 without data on T2DM; 4,074 with pre-existing T2DM; 2,121 who had a history of psychiatric illness (*n* = 1,024), ischemic heart disease (*n* = 638), or cerebrovascular disease (*n* = 496); 4,686 without data on overtime work; and 5,900 without data on covariates (smoking, *n* = 5,067; BMI, *n* = 84; and hypertension, *n* = 1,105). Some participants met more than one of these criteria for exclusion. Lastly, we excluded a further 3,307 workers who did not have any data at subsequent health checkups or who had no data needed to identify T2DM at all subsequent health exams. Thus, data on 33,050 workers (28,489 men and 4,561 women) aged 30–64 years (mean, 44.9; standard deviation [SD], 8.0 years) were included for analysis.

### Overtime work hours

Working hours were measured differently across the four participating companies as described previously^9^ and were integrated for main analysis into four categories from 1 (Short) to 4 (Long). Briefly, in one company, overtime working hours were assessed at each health checkup using a question with response options of: <45, 45–<80, 80–<100, or ≥100 hours per month, and no conversion was made for analysis. A similar question was used in another company: <45, 45–<60, 60–<80, 80–<100, or ≥100 hours per month in the last 2–3 months, and the categories of 45–59 and 60–<80 hours were integrated into category 2 (second lowest category) for main analysis. In the third company, workers self-reported overtime hours during the 1-month period of September, with 11 categories (from “0–10” to “>100 hours” per month), and 0 to 40 hours were converted to the category of 1 (Short), 41–80 hours to 2, 81–100 hours to 3, and >100 hours to 4 (Long). In the remaining company, average daily total working hours were self-reported at each health exam. We converted the data on daily working hours into monthly overtime with the formula: (daily working hours − 8) × 20 days as continuous data and then, classified the data on overtime into four categories (1 to 4).

### Biochemical measurements

Plasma glucose level was estimated using an enzymatic method or a glucose oxidase electrode method. Glycated hemoglobin (HbA1c) level was determined using high performance liquid chromatography method, latex agglutination immunoassay, or an enzymatic method. All laboratories performing these tests received high scores (score >95 out of 100 or rank A) by external quality control agencies.

### Type 2 diabetes mellitus

T2DM was diagnosed as a fasting plasma glucose of ≥7.0 mmol/L, a random plasma glucose of ≥11.1 mmol/L, HbA1c of ≥48 mmol/mol, or current treatment for diabetes. We defined incident cases of T2DM as those who met the diagnostic criteria at any examination after the baseline examination, until March 2014.

### Covariates

Body height and weight were measured based on a standard protocol in each of the participating company. We calculated body mass index (BMI) as weight (kg) divided by the squared height (m). Participants self-reported history of disease and health-related lifestyle factors using a questionnaire, the content of which differed among companies. Hypertension was diagnosed as systolic blood pressure of ≥140 mm Hg, diastolic blood pressure of ≥90 mm Hg, or current treatment for hypertension. Information on working condition, lifestyle habits, and family history of disease was obtained in one company and used for sensitivity analysis to adjust for these variables.

### Statistical analysis

Descriptive data according to overtime hours are shown as mean (SD) for continuous data and number (percentages) for categorical data. Participants were considered to be at risk for T2DM until the date of diagnosis of T2DM or the date of last examination during follow-up, whichever came first. We used Cox regression to calculate hazard ratios (HR) with 95% confidence intervals (CI) for T2DM. Linear trend was tested by assigning 23, 62, 90, and 120 to categories 1, 2, 3, and 4 of overtime, respectively. Model 1 was adjusted for company (four companies), age (continuous, years), and sex. Model 2 was additionally adjusted for BMI (continuous, kg/m^2^), smoking (never, past, or current), and factors in model 2 plus HbA1c level (continuous, mmol/mol) at baseline to create Model 3.

In one company, where data on working conditions; lifestyle, including sleep habits; and family history of T2DM were available (*n* = 27,590), we adjusted for sex and age (continuous, year) as Model 4. Alcohol use (non-drinker or drinker consuming <1, 1–2, and ≥2 *go* of Japanese sake equivalents a day [1 *go* of Japanese sake contains approximately 23 g of ethanol]), smoking status (never, past, or current), occupational physical activity (sedentary, standing or walking, or physically fairly active), department type (field work or non-field work), shift work (yes or no), job position (high or low), family history of T2DM (yes or no), and hypertension (yes or no) at baseline were additionally adjusted for Model 5. Sleep duration (<5.0, 5.0–5.9, 6.0–6.9, and ≥7 hours a day) and exercise during leisure (<2.5 or ≥2.5 hours a week) at baseline were further adjusted for in Model 6. In Model 7, baseline HbA1c (continuous) was additionally adjusted.

Participants were classified into four groups according to overtime work (<45 or ≥45 hours) and sleep duration (<5 or ≥5 hours); the group with <45 hours of working overtime and ≥5 hours of sleep was used as the reference group. We examined potential effect modification by sleep on the association of overtime work with T2DM in the fully adjusted model using a likelihood ratio test comparing models with and without interaction terms. All *P* values are two-sided, and *P* values <0.05 were considered statistically significant. We performed all analyses with Stata statistical software, ver. 14.2 (StataCorp, College Station, TX, USA).

## RESULTS

Baseline characteristics of participants are shown according to overtime work category in Table 1. Participants with longer working hours tended to be male, younger, and had a higher BMI but a lower proportion of hypertension. HbA1c level and smoking prevalence were not materially different according to overtime work category.

**Table 1.** Baseline characteristics of participants according to monthly overtime working hours

	Categories of overtime work (hours per month)			

	1 (Short)	2	3	4 (Long)
4 companies				
Number of participants	23,012 (69.9)	8,217 (25.0)	1,205 (3.7)	476 (1.5)
Sex, male	18,684 (81.2)	8,035 (97.8)	1,189 (98.7)	469 (98.5)
Age, years	45.6 (8.2)	43.4 (7.3)	42.9 (7.0)	43.3 (6.7)
BMI, kg/m^2^	23.3 (3.3)	23.5 (3.1)	23.7 (3.1)	23.6 (3.1)
HbA1c, %	5.6 (0.3)	5.6 (0.3)	5.6 (0.3)	5.6 (0.3)
Hypertension	4,156 (18.1)	1,046 (12.7)	137 (11.4)	48 (10.1)
Smoking	9,115 (39.6)	3,246 (43.2)	461 (38.3)	181 (38.0)

BMI, body mass index; HbA1c, glycated hemoglobin.

Data are shown as mean (SD) for continuous variables and number (percentages) for categorical variables. Overtime working hours were measured differently across the four participating companies and were categorized into the categories of 1 (Short) to 4 (Long). Briefly, in 3 companies, <45 hours as category 1, 45–<80 hours as category 2, 80–<100 hours as category 3, and ≥100 hours as category 4; in another company, <40 hours as category 1, 41–80 hours as category 2, 81–100 hours as category 3, and >100 hours as category 4.

During a mean follow-up period of 4.5 years, T2DM occurred in 1,975 participants. In all models, overtime hours were not materially associated with an increase in the risk of T2DM (Table 2). For example, compared with individuals with short overtime work (category 1), the HR of T2DM was 0.94 (95% CI, 0.64–1.38) for those with long overtime work (category 4) (Model 3, *P* for trend = 0.97). In the fully adjusted model, sleep duration was associated with T2DM risk in a U-shaped manner (*P* for quadratic trend = 0.036). As compared with sleeping 6–<7 hours per day, the HR was 1.18 (95% CI, 0.97–1.45) for sleeping <5 hours per day.

**Table 2.** Association of overtime working hours with risk of type 2 diabetes among Japanese workers

	Categories of overtime work (hours per month)				*P* for trend^a^

	1 (Short)	2	3	4 (Long)	
Four companies					
Number of cases	1,362	472	71	27	
Number of subjects	23,012	8,217	1,205	476	
Person-years	101,777	38,383	5,627	2,189	
Model 1^b^	1.00 (reference)	0.95 (0.86, 1.06)	0.95 (0.75, 1.20)	0.91 (0.62, 1.33)	0.33
Model 2^c^	1.00 (reference)	1.00 (0.90, 1.11)	1.03 (0.81, 1.31)	0.99 (0.67, 1.45)	0.95
Model 3^d^	1.00 (reference)	0.99 (0.88, 1.10)	1.07 (0.85, 1.36)	0.94 (0.64, 1.38)	0.97
One company^e^					
Number of cases	1,092	461	60	23	
Number of subjects	18,265	7,837	1,010	410	
Person-years	82,857	36,763	4,749	1,930	
Model 4^f^	1.00 (reference)	0.96 (0.86, 1.08)	1.00 (0.77, 1.29)	0.94 (0.62, 1.42)	0.57
Model 5^g^	1.00 (reference)	1.05 (0.94, 1.18)	1.17 (0.90, 1.52)	1.07 (0.71, 1.62)	0.22
Model 6^h^	1.00 (reference)	1.04 (0.93, 1.17)	1.13 (0.87, 1.48)	1.02 (0.67, 1.55)	0.38
Model 7^i^	1.00 (reference)	1.02 (0.91, 1.14)	1.15 (0.88, 1.50)	0.94 (0.61, 1.43)	0.64

Data are shown as hazard ratio (95% confidence intervals).

^a^*P* for trend was calculated by assigning 23, 62, 90, and 120 to increasing categories of overtime work with treating this variable as continuous one.

^b^Adjusted for age (years, continuous), sex, and worksite (4 companies).

^c^Additionally adjusted for smoking (never, past, or current), body mass index (kg/m^2^, continuous), and hypertension (yes or no).

^d^Further adjusted for HbA1c (%, continuous).

^e^Overtime work was measured using a question with 5 response options: <45, 45–<60, 60–<80, 80–<100, and ≥100 hours per month.

^f^Adjusted for age (continuous, year) and sex.

^g^Adjusted for baseline factors including age, sex, smoking (never, past, or current), alcohol consumption (non-drinker or drinker consuming <1, 1–2, and ≥2 *go* of Japanese sake equivalents per day), occupational physical activity (sedentary, standing or walking, and physically fairly active), shift work (yes/no), type of department (field-work related or not), job position (high/low), family history of diabetes (yes/no), and hypertension (yes/no).

^h^Adjusted for factors in model 6 plus leisure-time exercise (<150 or ≥150 min per week) and sleep duration (<5.0, 5.0–5.9, 6.0–6.9, and ≥7 hours per day) at baseline.

^i^Further adjusted for baseline HbA1c (continuous, mmol/mol).

**Figure 1.**
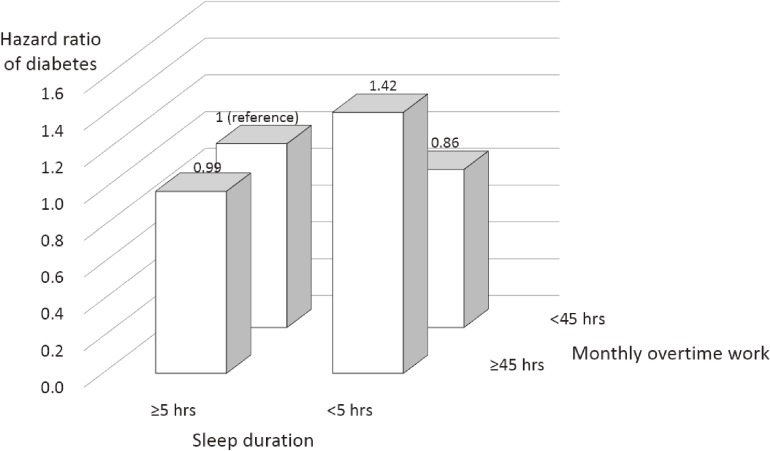
Combined association of overtime work and sleep duration with risk of type 2 diabetes. Data were adjusted for baseline variables, including age, sex, smoking, alcohol consumption, leisure-time exercise, occupational physical activity, shift work, job position, type of department, family history of diabetes, hypertension, body mass index, and HbA1c. HbA1c, glycated hemoglobin.

Figure 1 shows the combined association of overtime work and sleep duration with T2DM in one company. After adjustment for all covariates, including baseline HbA1c, long overtime (≥45 hours) combined with short sleep duration (<5 hours) was associated with a significantly higher risk of T2DM (HR 1.42; 95% CI, 1.11–1.83), whereas long overtime without sleep deprivation was not (HR 0.99; 95% CI, 0.88–1.11), both compared with monthly overtime of <45 hours and ≥5 hours sleep per day (*P* for interaction = 0.008).

## DISCUSSION

We found that overtime work was not associated with increased risk of T2DM. However, long working hours combined with short sleep duration were associated with a significantly higher T2DM risk, whereas individuals with long working hours but without sleep deprivation were not. This is one of the few investigations to address the association of working hours with T2DM incidence in Asia, and the first to report on the effect modification of sleep duration, globally.

Our finding of no association between overtime work and T2DM risk is supported by a meta-analysis using data of cohort studies predominantly in Europe and the United States,^3^ which showed a risk ratio of 1.07 (95% CI, 0.89–1.27) for working 55 hours per week (approximately 60 hours per month of overtime). In Asia, a cohort study of Japanese civil servants also reported no association with hyperglycemia.^6^ The observed lack of association may be related to the healthy worker effect. Healthy workers, who are at low risk of developing diseases including T2DM, might have worked long hours, resulting in the null finding. In contrast, previous Japanese studies^4^^,^^5^^,^^7^ showed the opposite findings. Specifically, two Japanese studies reported an inverse association in non-shift workers^5^ and white collar workers^7^ and a positive association in shift workers^5^ and blue collar workers.^4^ Given the inconsistent findings, no confident conclusion can be drawn for the effect of working long hours on glucose metabolism in Asian populations.

We observed that long overtime working hours combined with sleep deprivation showed a higher T2DM risk, whereas long working hours with enough sleep did not. Long working hours may cause sympathetic overactivation,^12^ which leads to hyperglycemia.^13^ In contrast, sufficient sleep is important to inhibit sympathetic activation.^14^ For individuals working long hours, sufficient sleep may be important to recover to a healthy level, whereas insufficient sleep may accentuate the sympathetic overactivation caused by overtime work.

This study has some strengths, including a large sample size, investigation of the effect of extremely long hours of working, and the longitudinal study design. However, limitations should be mentioned. First, working hours were not assessed uniformly across participating companies. Nonetheless, observed associations were not largely different between companies with sufficient numbers of individuals who worked overtime (data not shown). Second, data on working hours and sleep duration were assessed using self-report, so they might be inaccurate to some extent. If random misclassification occurred in these variables, the actual risk associated with overtime work and sleep duration would be higher than observed. Nonetheless, we confirmed that the present questionnaires on overtime or daily working hours are similar to the highly valid and moderately reproducible questionnaires among Japanese employees from the participating companies of J-ECOH Study.^15^ Therefore, the possibility of underestimation would be low. Third, in this study, reference category of overtime work was not no overtime work (0 hours); some participants with short overtime work (eg, >0 to <45 hours) may have been included in that category. Thus, if short overtime work may elevate diabetes risk, the risk associated with long overtime work might have been underestimated. Fourth, only baseline data were used for overtime work. Random changes in working hours during follow-up might have skewed the results toward the null. Fifth, in the participating companies, retirement age was generally set as 60 years, and those who retired at age 60 years may be rehired up to the age 65 years. Thus, workers aged 60 years or older at the entry were excluded mainly due to no follow-up data, potentially leading to biased results. However, exclusion of workers aged 60 years (*n* = 875) did not change the findings (data not shown). Sixth, unmeasured confounders, including socioeconomic status, and residual confounding might have affected the results. Nonetheless, in one company, adjustment for wide array of potential confounders did not change the findings substantially. Lastly, the participants worked in large-scale companies; the present findings may not be applicable to workers in companies with different background, including small- to medium-sized companies.

This study of Japanese workers from large-scale companies revealed that overtime work was not associated with an increase in T2DM risk. However, long overtime work was associated with an increased risk of T2DM among those who slept short hours. Further investigations are needed to clarify the long-term effect of long working hours on glucose metabolism and the modification of this effect by sleep deprivation.
